# Cationic Covalent Organic Framework with Ultralow HOMO Energy Used as Scaffolds for 5.2 V Solid Polycarbonate Electrolytes

**DOI:** 10.1002/advs.202200390

**Published:** 2022-05-26

**Authors:** Jie Liu, Yuhao Zhang, Haoqing Ji, Jing Zhang, Pinxin Zhou, Yufeng Cao, Jinqiu Zhou, Chenglin Yan, Tao Qian

**Affiliations:** ^1^ School of Chemistry and Chemical Engineering Nantong University Nantong 226019 China; ^2^ Key Laboratory of Core Technology of High Specific Energy Battery and Key Materials for Petroleum and Chemical Industry College of Energy Soochow University Suzhou 215006 China; ^3^ State Key Laboratory of Space Power‐sources Technology Shanghai Institute of Space Power‐Sources Shanghai 200245 China

**Keywords:** cationic covalent organic framework, high decomposition voltage, lithium metal battery, solid polymer electrolyte

## Abstract

Solid polymer electrolytes (SPEs) have become promising candidate to replace common liquid electrolyte due to highly improved security. However, the practical use of SPEs is still restricted by their decomposition and breakage at the electrode interfacial layer especially at high voltage. Herein, a new cationic covalent organic framework (COF) is designed and synthesized as a reinforced skeleton to resist the constant oxidative decomposition of solid polycarbonate electrolyte, which can stabilize cathode electrolyte interphase layer to develop long‐term cycle solid lithium metal battery. The ultralow HOMO energy (−12.55 eV according to density functional theory (DFT) calculations), reflecting its oxidation resistance at positive potential, would be responsible for the high decomposition voltage of 5.2 V versus Li^+^/Li of solid polycarbonate electrolyte. Furthermore, the smooth surface of interfacial layer and inhibited decomposition reaction at cathode side is confirmed in solid LiCoO_2_ cell, which realizes high initial capacity up to 160.3 mAh g^−1^ at 0.1 C and greatly improved stability in 4.5 V class solid polymer lithium metal battery with high capacity retention over 200 cycles. This new type of high‐voltage resistant solid polymer electrolyte promotes the realization of high‐voltage cathode materials and higher energy density lithium metal battery.

## Graphical abstract

A cationic covalent organic framework (COF) with strong antioxidant capacity is synthesized and used as high voltage resistant skeleton material in solid polycarbonate electrolyte. The stable COF acts as a reliable scaffold to prevent the collapse of cathode electrolyte interface (CEI) and the persistent decomposition of polycarbonate and the side reactions at electrode/electrolyte interface can be well restrained.

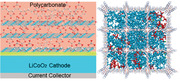

## 1 Introduction

Currently, lithium ion batteries (LIBs) have been extensively used in modern society, including energy storage, portable electronic devices, and electronic vehicles.^[^ ^1^, ^2^, ^3^ ^]^ With the urge to develop new battery system with higher energy density, lithium metal becomes the promising candidate to replace the graphite anode used in LIBs due to its high theoretical capacity (3860 mAh g^−1^) and low potential (−3.04 vs standard hydrogen electrode).^[^ ^4^ ^]^ Nonetheless, with the increase of energy density, the risk of combustion and even explosion of lithium metal battery (LMBs) is increasing since the widely used liquid organic electrolyte is volatile and extremely flammable.^[^ ^5^, ^6^ ^]^ It is an effective way to improve the safety by replacing liquid electrolyte with nonflammable solid electrolyte. Furthermore, solid electrolyte with higher modulus can inhibit growth of dendrites and reduce the risk of short circuit. Thanks to better compatibility with electrodes and higher ionic conductivity in solid polymer electrolytes (SPEs), polycarbonate‐based solid electrolytes have been widely studying over the years and have good application perspective.^[^ ^7^, ^8^, ^9^, ^10^, ^11^, ^12^, ^13^, ^14^ ^]^ Due to the high highest occupied molecular orbital (HOMO) level, valence electrons of polycarbonate which cannot be effectively restricted are prone to interact with lithium ions, leading to the inevitable side reactions at electrode surface and the formation of cathode electrolyte interface (CEI) layer.^[^ ^15^ ^]^ Unfortunately, for polycarbonate electrolyte, the CEI layer has been demonstrated to be unstable and polycarbonate electrolytes were constantly consumed during repeated charge–discharge cycle.^[^ ^16^, ^17^, ^18^ ^]^

The energy density of a battery has strong correlation with its voltage platform. As a result, besides employing lithium metal anode, to increase the energy density of batteries, it is essential to use high voltage cathode materials. The long term cycling performance of high‐voltage solid battery is closely linked to the properties of solid electrolyte and also CEI, which was first suggested on the surface of LiCoO_2_ by Goodenough and co‐workers.^[^ ^19^ ^]^ However, due to the inevitable volume change of cathode material during charge and discharge process, the CEI is easy to break particularly at high voltage and the electrolyte–electrode interface will be deteriorated with prolonged cycles, which is very different from the liquid batteries, where the CEI will inevitably crack and reform while electrolyte is constantly consumed with irreversible extraction of Li^+^ ions from the cathode.^[^ ^20^, ^21^ ^]^ Such effects will be exacerbated in following cycles and greatly shorten battery life. An outstanding CEI layer should be electrochemically stable to isolate electrolyte from high voltage cathode and have high mechanical property to stand the volume change of cathode material during cycling.^[^ ^17^, ^22^, ^23^, ^24^ ^]^ Therefore, a good antioxidizing SPE which is stable even at a higher operation voltage and enables the formation of a robust CEI will be favorable for achieving long‐term cycle stability of high voltage cathodes.

Some organic framework materials, such as covalent organic frameworks (COFs) and metal organic frameworks (MOFs), nowadays attract much attentions due to their porous and crystalline features that assembled by the stable linkage with in‐built functionality. Their tunable structure, porosity and high specific surface area lead to the applications in solar cell, catalysis, and electrolyte field.^[^ ^25^, ^26^, ^27^, ^28^, ^29^, ^30^, ^31^ ^]^ COFs generally have more stable structure and high designability and thus are popular in the researching of rechargeable lithium metal batteries. Bu and co‐workers applied Tb‐DANT‐COF as electrode materials and achieved high initial capacity of 144 mAh g^−1^, which can be assigned to the multiple redox active sites.^[^ ^32^ ^]^ Horike and co‐workers incorporated poly(ethylene oxide) (PEO) chains into the inner space of 2D COFs and prepared COF‐based solid electrolyte, which provided the fast Li^+^ ion transportation channels from the continuous and stable porous framework.^[^ ^33^ ^]^

Herein, inspired by traditional reinforced concrete structure, we propose a reinforced skeleton strategy to form stable CEI scaffold and avoid persistent decomposition of polycarbonate by introducing high voltage resistant cationic COF material into polycarbonate. A cationic COF with ultralow HOMO value of −12.55 eV, which has strong antioxidant capacity, was designed and synthesized as high voltage resistant skeleton material. After introducing antioxidative cationic COF into solid polycarbonate electrolyte (C‐SPE), the stable COF acts as a reliable scaffold to prevent the collapse of CEI and the persistent decomposition of polycarbonate and the side reactions at electrode/electrolyte interface can be well restrained. As a result, a significantly enhanced CEI with higher modulus and stability was formed compared with pristine solid polycarbonate electrolyte (P‐SPE). Moreover, molecular dynamic (MD) simulations demonstrate the dissociation of lithium ions is promoted while the migration of anion is inhibited. The high Li^+^ conductivity of 1.3 × 10^−4^ S cm^−1^ at 25 °C and Li^+^ transfer number up to 0.62 are measured. The assembled solid LiCoO_2_ cell achieves initial capacity of 160.3 mAh g^−1^ (0.1C) and greatly improved capacity retention of 83.9% at 1C after 200 cycles. This work provides a new strategy to improve the electrochemical stability window of SPE and enable higher energy density lithium metal battery with high‐voltage cathode.

## 2 Result and Discussion

**Figure 1:**
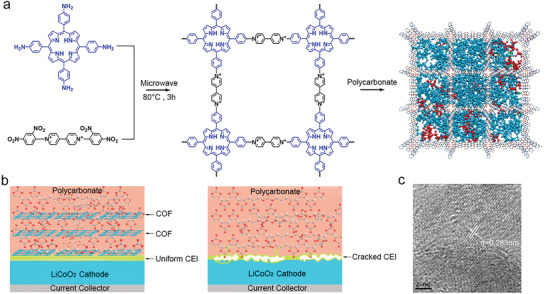
a) Synthetic procedure of COF samples and the schematic of C‐SPE. b) CEI growth diagram of solid LiCoO_2_ battery with C‐SPE and P‐SPE. c) An HRTEM image of COF (interplanar distance = 0.283 nm, Scale bar: 2 nm).

The cationic COF was firstly synthesized by the Zincke reaction between 1,1′‐bis(2,4‐dinitrophenyl‐4,4′‐bipyridin)‐1,1′‐diium dichloride and 5,10,15,20‐Tetrakis(4‐aminophenyl) porphyrin in an ethanol/water (1:1 v/v) solution under microwave heating at 100 °C for 3 h.^[^ ^34^, ^35^ ^]^ C‐SPE is prepared by adding COF samples into poly(diallyl carbonate‐vinyl carbonate), e.g., PDV, matrix (**Figure** **1**a). The introduction of COF into polycarbonate is aimed at stabilizing the CEI layer in Li|C‐SPE|LiCoO_2_ cell (Figure 1b). As shown in Figure S1 (Supporting Information), the disappearance of the stretch vibration of C═C bonds in vinyl carbonate and diallyl carbonate at 1538 cm^−1^ confirms their polymerizations. Figure S2 in the Supporting Information shows the solid‐state ^13^C NMR spectra of COF with the marked peaks corresponding to the four types of carbon atoms of COF network. The X‐ray photoelectron spectroscopy (XPS) of COF are shown in Figure S3 in the Supporting Information. The lattice image indicates the well crystallinity of COF. From the N1s spectra, the characteristic peaks at 401.5, 399, and 397 eV are attributed to C–N^+^, C–N, and unprotonated pyridinic sites of porphyrin, respectively.^[^ ^36^, ^37^ ^]^ Figure 1c shows the interplanar distance of COF lattice by high resolution transmission electron microscope (HRTEM). The typical image of in situ polymerized C‐SPE at 80 °C for 24 h are shown in Figure S4a,b (Supporting Information) which indicates that the homogeneous precursor after solution ultrasonic dispersion can transform into uniform solid electrolyte after polymerization. Figure S4c,d (Supporting Information) shows the scanning electron microscope (SEM) image of the uniform surface of C‐SPE from the top‐view and cross‐section. The molecular weight of PDV (*M*_n_: 237 589, *M*_w_: 442 424) is characterized by gel permeation chromatography (GPC) as shown in Figure S5 in the Supporting Information.

**Figure 2:**
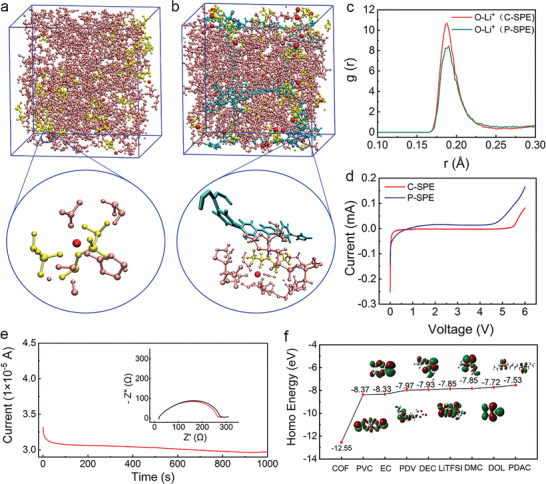
Conformation analysis of the system with a) P‐SPE, b) C‐SPE from MD simulations. Li‐ion: red; TFSI^−^: yellow; Polycarbonate: pink; COF: cyan. c) Li‐ion radial distribution functions in P‐SPE and C‐SPE. d) LSV curves of P‐SPE and C‐SPE. e) *t*_+_ of C‐SPE. Insert is EIS curves of before (red) and after (black) polarization. f) HOMO energy of EC, DEC, LiTFSI, DMC, DOL, and the structural units of cationic COF, poly(vinyl carbonate) (PVC), poly(diallyl carbonate) (PDAC), and PDV.

MD simulations for whole SPE system including COF, polycarbonate and LiTFSI are performed aimed at evaluating the lithium ion conductivity of C‐SPE. **Figure** **2**a,b displays the conformation analysis of the system with P‐SPE and C‐SPE, respectively. Polycarbonate is uniformly distributed in the box. For P‐SPE as shown in the enlarged spatial distribution in Figure 2a, lithium ion coordinates evenly with polycarbonate electrolyte and TFSI^−^. Meanwhile, Figure 2b illustrates the TFSI^−^ anions tend to be distributed near the COF structure, and the lithium ions dissociated from LiTFSI are likely to coordinate with polycarbonate segment. The radial distribution functions (RDF) value of Li–O, representing the probability that finding Li atom in a shell d*r* at the distance *r* of O atom chosen as reference point, in C‐SPE increase obviously compared with that of P‐SPE as demonstrated in Figure 2c. This indicates that the affinity between lithium ion and polycarbonate segment is enhanced, which can increase the lithium ion conductivity in SPE. The calculated self‐diffusion coefficient of TFSI^−^ in C‐SPE and P‐SPE is about 1.523 × 10^−9^ and 8.654 × 10^−9^ cm^2^ s^−1^ respectively according to the GROMACS software with default options. The difference of TFSI^−^ diffusion coefficient is attributed to the ion‐mobility inhibition by cationic COF. To confirm the evaluation of MD simulations, the Li^+^ conductivities of P‐SPE and C‐SPE were measured by the alternating current (AC) impedance. The ionic conductivity values of P‐SPE with the different weight ratios of VC/DAC are listed in Table S1 in the Supporting Information. The ratio of VC/DAC = 75/25 wt% is aimed to achieve the highest ionic conductivity after polymerization into solid electrolyte. The ionic conductivities were measured to be 5.8 × 10^−5^, 6.3 × 10^−5^, and 7.2 × 10^−5^ S cm^−1^ as indicated in Table S1 (Supporting Information), corresponding to the VC/DAC weight ratios of 5:1, 4:1, and 3:1, respectively. However, when the ratio was further improved to 2:1, the electrolyte cannot be polymerized into solid, which can be ascribed to the situation that the monomer ratio has important effect on the polymerization reaction and properties of polymers. As a result, the ratio of VC/DAC was determined to be 75/25 wt%. Figure S6 (Supporting Information) showed the Li^+^ conductivity‐temperature curve which agrees well with the Arrhenius law. The ion conductivity of C‐SPE and P‐SPE are measured to be 1.3 × 10^−4^ and 7.2 × 10^−5^ S cm^−1^ at room temperature, respectively. The Li ion transform numbers (*t*_+_) of two SPEs are also evaluated and the *t*_+_ value of P‐SPE is 0.39 (Figure S7, Supporting Information) at room temperature but increased to 0.62 (Figure 2e) for C‐SPE. This promotion fits well with the MD simulation, which shows the inhibited diffusion of TFSI^−^ and promoted Li^+^ diffusion of lithium ion diffusion in C‐SPE.

To better evaluate the high voltage resistant performance of C‐SPE, the oxidation potential of electrolyte is analyzed by the calculated HOMO value carried out by the density functional theory (DFT).^[^ ^38^, ^39^, ^40^ ^]^ Figure 2f shows the HOMO level of some electrolyte materials or structural units of poly(vinyl carbonate), poly(diallyl carbonate), PDV, and COF. The calculation results demonstrate that compared with electrolyte components, cationic COFs have much higher oxidation resistance, which indicate that COFs have better electrochemical stability even under high voltage on the cathode side. The detailed structural units of PDV and cationic COF used in DFT calculations are shown in Figure S8 in the Supporting Information. The cationic COFs act as reinforced skeletons in the formation of CEI and thus improve the electrochemical stability. To verify the DFT calculation, the electrochemical stability windows of C‐SPE and P‐SPE are evaluated by the linear scanning voltammetry (LSV) measurements. As shown in Figure 2d, the P‐SPE shows the decomposition potential stability up to 4.2 V versus Li^+^/Li. The electrochemical stability of the electrolyte is enhanced when the amount of COF increases. The electrochemical stable window of electrolyte is increased to 5.2 V when the COF additive is increased from 10 to 30 mg mL^−1^ as shown in Figure S9 in the Supporting Information. There is no obvious window increase when the amount of COF was further increased. As a result, the amount of COF is reasonably chosen to be 30 mg mL^−1^.

**Figure 3:**
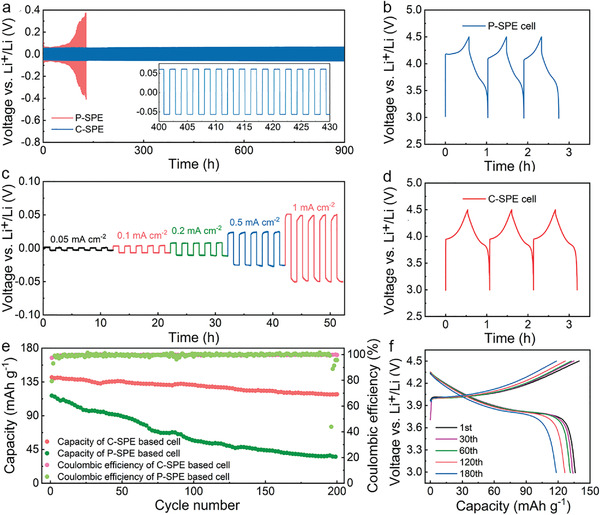
a) The voltage profiles of lithium plating and stripping in Li|Li cell with P‐SPE and C‐SPE at 1 mA cm^−2^ under room temperature. Insert is the enlarged voltage profile after 400 h. b) The voltage profiles of LiCoO_2_|P‐SPE| Li cell during the initial three cycles. c) The voltage profiles of lithium plating and stripping in Li|Li cell with C‐SPE at different current density. d) The voltage profiles of LiCoO_2_|C‐SPE|Li cell during the initial three cycles. e) Cycling performance of LiCoO_2_|P‐SPE|Li cell and LiCoO_2_|C‐SPE|Li at the current density of 1C (Pink: Coulombic efficiency of C‐SPE based cell. Green: Coulombic efficiency of P‐SPE based cell. Red: Capacity of C‐SPE based cell. Dark green: Capacity of P‐SPE based cell). f) The charge–discharge curves of LiCoO_2_|C‐SPE|Li cell at different cycles.

The polarization performance is measured to evaluate the reversibility of long‐term Li deposition/stripping cycles. The **Figure** **3**a shows the stable cycling of Li|C‐SPE|Li symmetry cells for 900 h under 1 mA cm^−2^. Meanwhile, the sharp voltage fluctuation appears at 128 h, which indicates short circuit caused by the decomposition and growth of dendrite. The bad performance of Li|P‐SPE|Li symmetry cells with high overpotential implies the unstable interfacial layer. The difference of the polarization performance between C‐SPE and P‐SPE is mainly due to the high Li‐ion conductivity and modulus of C‐SPE. Figure S10a,b (Supporting Information) displays the modulus of P‐SPE is ≈3.4 GPa, while the one of C‐SPE is increased to 4.6 GPa after the reinforcement of COFs. Figure S10c,d (Supporting Information) shows the modulus maps of P‐SPE and C‐SPE, respectively. The Li deposition/stripping cycling performances of lithium symmetry cells (Li|C‐SPE|Li) at the current density of 0.05, 0.1, 0.2, 0.5, and 1 mA cm^−2^ are shown in Figure 3c. The polarization voltage is as low as 1 (0.05), 3 (0.1), 7 (0.2), 23 (0.5), and 52 mV (1 mA cm^−2^) at room temperature. The Li deposition/stripping behavior at different current densities for P‐SPE are tested for comparison. As shown in Figure S11 (Supporting Information), when the current density is over 0.2 mA cm^−2^, the polarization voltage of P‐SPE become fluctuate and much higher than that of C‐SPE.

Galvanostatic charge and discharge are measured to evaluate the electrochemical performance of the coin LiCoO_2_|C‐SPE|Li and LiCoO_2_|P‐SPE|Li cells at room temperature. Figure 3b,d displays their voltage profiles of initial three cycles at the rate of 1C. The charge and discharge profiles in Figure 3b show poorer symmetry than Figure 3d, which indicates the worse reversibility and lower initial coulombic efficiency of P‐SPE based cell than that of C‐SPE based cell. Compared with LiCoO_2_|P‐SPE|Li cell, which exhibits rapid capacity loss and unsatisfactorily cycling stability, the LiCoO_2_|C‐SPE|Li displays the greatly improved capacity retention of 83.9% at 1C after 200 cycles and the coulombic efficiency is up to 99.8% as shown in Figure 3e. In addition, the rate performance of LiCoO_2_ cell based on both C‐SPE and P‐SPE are compared as seen in Figure S12 (Supporting Information), which demonstrates the LiCoO_2_|P‐SPE|Li cell exhibits much lower rate capacities and faster capacity decay than LiCoO_2_|C‐SPE|Li cell. The LiCoO_2_|C‐SPE|Li cell has an initial capacity of 160.3 mAh g^−1^ at 0.1C in Figure S13 in the Supporting Information. In Figure 3f, the charge–discharge curves of C‐SPE cell at 1st, 30th, 60th, 120th, and 180th cycle exhibits high reversibility and stable discharge plateaus. The morphologies of lithium anodes retrieved from two cells after 200 cycles are characterized by SEM (Figure S14, Supporting Information). The SEM image of lithium metal in P‐SPE based cell shows obvious cracks and dendrites. In contrast, the lithium metal in C‐SPE based cell is smooth, which suggests the good inhibition of dendrites. The growth of dendrite at lithium metal anode is mainly influence by the electric field and charge distribution. Apart from increasing the modulus, reducing the space‐charge region close to Li metal is also efficient to ensure the uniform electrodeposition of lithium. The immobilized anion by the cationic COF in C‐SPE is essential to the inhibition of dendrite at Li metal anode. The high reversibility and good lithium plating and stripping performance of C‐SPE cell demonstrate the C‐SPE is a promising electrolyte for the new type of solid batteries with high energy density.

**Figure 4:**
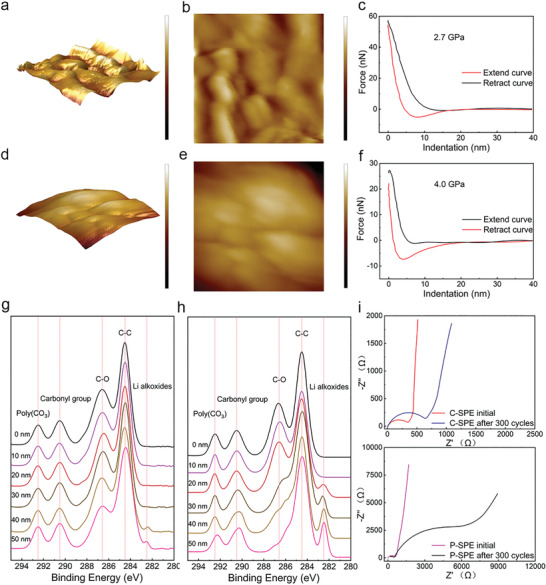
a) AFM 3D images (scan size: 0.5 × 0.5 µm^2^, scale bar: −100 to 50 nm), b) AFM images (scan size: 0.5 × 0.5 µm^2^, scale bar: −100 to 50 nm), and c) the indentation curves (Black: Extend curve, Red: Retract curve) of cycled CEI layer in LiCoO_2_|P‐SPE|Li after 200 cycles at the current density of 1C. d) AFM 3D images (scan size: 0.5 × 0.5 µm^2^, scale bar: −100 to 50 nm), e) AFM images (scan size: 0.5 × 0.5 µm^2^, scale bar: −100 to 50 nm), and f) The indentation curves (Black: Extend curve, Red: Retract curve) of cycled CEI layer in LiCoO_2_|C‐SPE|Li cell after 200 cycles at the current density of 1C. The XPS sputtering spectra of g) CEI layer in C‐SPE based cell and h) CEI layer in P‐SPE based cell with the depth from 0 to 50 nm. i) The initial and cycled EIS of C‐SPE cell and P‐SPE based cell.

Atomic force microscope (AFM) images of CEI layer are measured to evaluate the mechanical strength and interfacial morphology of P‐SPE and C‐SPE based CEI layer. The measurement was performed on the disassembled P‐SPE and C‐SPE based cells after 200 cycles. The 3D AFM image of the CEI layer in P‐SPE based cell fluctuates at a large scale and forms a rough interface. **Figure** **4**a,b shows the uneven interface with obvious cracks due to the severe decomposition of electrolyte and low CEI strength. Unlike P‐SPE based cell, Figure 4d,e displays the uniform CEI layer in C‐SPE based cell. The continuous smooth feature demonstrates the good interfacial compatibility and electrochemical stability of CEI layer. Figure 4c,f shows the force curve as a function of indentation. The intersection of the extension line applying the external thrust and the curve of the free position is set to the zero point of indentation.^[^ ^41^, ^42^ ^]^ The modulus of CEI in P‐SPE cell is ≈2.7 GPa, which was enhanced to ≈4.0 GPa in C‐SPE based cell. The improvement of modulus contributes to the stability of CEI layer.

XPS sputtering measurement was performed on the disassembled LiCoO_2_ cells after 200 cycles at the current rate of 1C in order to analyze the composition of the CEI layer. Figure 4g exhibits the XPS sputtering spectrum of CEI layer of C‐SPE electrolyte cell with the increasing sputtering depth from 0 to 50 nm. In C1s XPS spectrum of the CEI cycled in C‐SPE, five distinctive peaks appear at 292.5, 290.5, 286.6, 284.5, and 282.5 eV, which are attributed to polycarbonate (poly(CO_3_)), carbonyl group, ether oxygen, carbonate group, and Li alkoxides (ROLi) respectively.^[^ ^43^, ^45^ ^]^ The peak intensity of ROLi is closely related to the decomposition of polycarbonate due to the ring opening reaction during the charge–discharge process.^[^ ^46^ ^]^ In the spectra of C‐SPE, only weak ROLi peak are measured at the depth 40 nm from initial position. On the contrast, the XPS sputtering spectrum (Figure 4h) of CEI cycled in P‐SPE show obvious and strong ROLi peak at the depth 20–50 nm from initial position. This strong peak is due to the violent decomposition of polycarbonate in P‐SPE. Moreover, Figure 4i displays the electrochemical impedance spectrum of before and after 200 cycles of LiCoO_2_|C‐SPE|Li and LiCoO_2_|P‐SPE|Li cells. For the C‐SPE based cell, the initial interface impedance and cycled interface impedance are close. Meanwhile, for the P‐SPE based cell, the charge‐transfer resistance increases to ≈6557 Ω, which corresponds to the rapid capacity loss of P‐SPE based cell after 200 cycles. Both the XPS sputtering measurements and EIS demonstrate the well‐formed interface layer of C‐SPE cell.

## 3 Conclusion

In this work, a new cationic COF with ultralow HOMO value of −12.55 eV, which has a strong antioxidant capacity was synthesized and employed as reinforced skeleton to inhibit the electrolyte from constant decomposition under high voltage. By introducing COF into polycarbonate electrolyte, the modulus and stability of resulted CEI layer are all improved, and the high voltage performance of solid lithium metal battery was improved. The cationic COFs inhibit the mobility of anions and promote the mobility of lithium ions, contributing to the improvement of lithium ion transfer number (0.62). The ion conductivity (1.3 × 10^−4^ S cm^−1^ at 25 °C) is improved due to extra lithium ion transport pathway originating from the porous structure of cationic COFs. As a result, the LiCoO_2_|C‐SPE|Li cell displays initial capacity of 160.3 mAh g^−1^ (0.1C) and the capacity retention is greatly improved to 83.9% at 1C after 200 cycles. The electrochemical performances of some solid polymer electrolytes are summarized in the Table S2 (Supporting Information) for comparison. The huge advantage of C‐SPE in the electrochemical stability window can be witnessed than conventional polymer electrolyte. Our study develops a new strategy of high‐voltage resistant solid polymer electrolyte and provides ideas for designing high energy density solid state lithium metal batteries with high‐voltage cathodes.

## Conflict of Interest

The authors declare no conflict of interest.

## Supporting information

Supporting Information

## Data Availability

The data that support the findings of this study are available from the corresponding author upon reasonable request.
