# Supplementary material for: Cationic Covalent Organic Framework with Ultralow HOMO Energy Used as Scaffolds for 5.2 V Solid Polycarbonate Electrolytes
Source: Adv Sci (Weinh). 2022 May 26;9(21):2200390. doi: 10.1002/advs.202200390 (PMC9313477; doi:10.1002/advs.202200390)
Supplement: Supplementary file 1 — Supporting Information [file ADVS-9-2200390-s001.pdf]

Supporting information

Cationic Covalent Organic Framework with Ultra-low HOMO Energy Used as Scaffolds for 5.2 V Solid Polycarbonate Electrolytes

Jie Liu, Yuhao Zhang, Haoqing Ji, Jing Zhang, Pinxin Zhou, Yufeng Cao, Jinqiu Zhou, Tao Qian,* and Chenglin Yan*

J. Liu, Prof. Y. Cao, J. Zhou, Prof. T. Qian

School of Chemistry and Chemical Engineering, Nantong University, Nantong 226019, China

E-mail: qiantao@ntu.edu.cn (T. Qian)

Y. Zhang, H. Ji, Y. Zheng, Prof. C. Yan

College of Energy, Key Laboratory of Advanced Carbon Materials and Wearable Energy Technologies of Jiangsu Province, Soochow University, Suzhou 215006, China

E-mail: c.yan@suda.edu.cn (C. Yan)

J. Zhang, P. Zhou

State Key Laboratory of Space Power-sources Technology, Shanghai Institute of Space Power-Sources, Shanghai 200245, China.

EXPERIMENTAL SECTION

Preparation of COF and C-SPE

The 1,1′–bis(2,4–dinitrophenyl)–(4,4′–bipyridin)–1,1′–diium dichloride (200 mg) and 5,10,15,20–Tetrakis(4–aminophenyl) porphyrin (166 mg) dissolved in an ethanol/water (5:5, v/v) mixture (100 mL) under microwave heating with 2.45 GHz using a microwave chemical reactor (MCR–3) at 100 °C for 3 hours. The product was filtered three times with deionized water (250 mL) and ethanol (250 mL) to remove the monomers. Then the sample is vacuum dried at 70 °C for 24 h to prepare the final production. Dissolving 1 M LiTFSI into monomer mixture of vinylene carbonate (VC, 75 wt%) and diallyl carbonate (DAC, 25 wt%) to prepare the precursor solution. The sufficient COF (30 mg) was added into precursor solution (1 mL) and then the solution was ultrasonically dispersed for 3 h using a high power ultrasonic cleaner (KQ–100KDB). Finally, the initiator AIBN (1 wt% of monomer) was added into the solution and the polymerization occurred completely after heating at 80 °C for 48 h.

Characterizations

Fourier Transform infrared spectroscopy (FTIR) studies were carried out on the BRUKER spectrometer in the range 2300 – 400 cm^{-1}. The FTIR spectra of COF is shown in Figure S1. Solid–State ^{13}C Nuclear Magnetic Resonance (NMR) tests were recorded on Bruker Avance spectrometer, with a working frequency of 400 MHz for ^{13}C nuclei. X–ray photoelectron spectroscopy (XPS) spectra were observed in an ultra–high vacuum ESCALAB 250 set–up equipped with a monochromatic Al Ka X–ray source (1486.6 eV; anode operating at 15 kV and 20 mA) and equipped with an Ar^{+} sputtering gun (Thermo Fisher). Ar^{+} etching was conducted at an argon partial pressure of 10^{-8} Torr in the x–y scan

mode at ion acceleration of 2 kV and ion beam current density of 1 mA mm^{-2}.

The morphology and size of COF was determined with a TEM (FEI TECNAI G2 F20) and high resolution TEM (FEI TECNAI G20). All samples were scattered on a carbon–coated copper grid. Scanning electron microscopy (SEM) images were measured using HITACH SU8010. The Young's modulus of sample was analyzed by atomic Force Microscope (AFM) Dimension Icon.

Electrochemical Measurements

The EIS measurement was carried out on CHI660E with the frequency range 1000 kHz to 0.1 Hz. Electrochemical stability window was determined with linear sweep voltammetry (LSV, CHI660E) utilizing Li metal|SPE|stainless (SS) cells. The lithium ion conductivity (σ) was calculated by the following equation:

$$\sigma = \frac{d}{RS}$$

Where d represents the thickness of C-SPE, R is the resistance obtained from EIS measurements, and S represents the working area of electrode. The Li–ion transform number (t_+) was calculated by following equation:

$$T_+ = \frac{I_s(\Delta V - T_0 R_0)}{I_0(\Delta V - T_s R_s)}$$

Where ΔV is the polarization voltage (10 mV), I_s and R_s represent the steady current and resistance value after polarization, and I_0 and R_s are initial current and resistance value. The polarization of SPE and the cycling performance of $LiCoO_2$|SPE|Li cells were measured utilizing the CT2001A cell test instrument (Wuhan LAND Electronic Co. Ltd).

Computational method

Density functional theory (DFT) describes the electronic properties based on the electron charge density of the system, and has been used widely in material simulations

studying systems from crystal to organic molecules. However, due to their high order scaling, the DFT method can only be applied to relatively small systems. For the polymer molecules, the large size system beyond the capability of the straight forward DFT calculations.[1] The large molecular weight of PDV (Mn: 237589, Mw: 442424) is confirmed by Gel Permeation Chromatography (GPC) in Figure S13. In this work, in view of the large size of PDV and the similar molecular environments of repeating unit, the structural unit of PDV, e. g. -$C_3H_2O_3$-$C_7H_{10}O_3$-, is used to calculate the HOMO. The detailed sructural units of PDV, poly(vinylene carbonate), poly(diallyl carbonate) and cationic COF used in DFT calculations are shown in Figure S14. The HOMO computations were carried out using rb3lyp density functional method. All the atoms in the geometry optimizations uses the 6–31G(d+p) basis set. The vibration frequency of all optimized structures is analyzed at the same theoretical level. The Gaussian 0937 suite of programs was used throughout. The HOMO of EC, DEC, LiTFSI, DMC, DOL, and the structural units of cationic COF, poly(vinyl carbonate), poly(diallyl carbonate) and PDV were computed to measure the decomposition potential.

MD simulations were carried out using Gromacs, v5.0.5. The object system of BSPE consists of 29 PDV, 33 LiTFSI. Meanwhile, while two extra COF molecules were added into the object system of BSPE. PDV was modeled by the OPLS–AA force field,[2] with the parameters generated by the LigParGen server.[3] LiTFSI was modeled according to Sun et al.[4] COF was modeled by UFF force field,[5] with the atomic charges derived by the Multiwfn program after geometry optimization at the DFT/B3LYP level of theory with the cc–pVDZ basis set implemented in Gaussian 09 software package.[6] Interatomic interactions were described by the Van der Waals (VDW) interactions and coulombic interactions. The VDW

interactions were expressed by the Lennard Jones (LJ) potential truncated at 1.2 nm. LJ parameters of different atom pairs were generated by geometric combination rules, i.e., $\sigma_{ij}=(\sigma i\sigma j)^{1/2}$ and $\varepsilon_{ij}=(\varepsilon i\varepsilon j)^{1/2}$. The coulombic interactions were calculated by the particle mesh Ewald algorithm., with the short–range part truncated at 1.2 nm, and the long–range part calculated in the reciprocal space with a Fourier spacing of 0.12 nm. The leap–frog algorithm with a time–step of 1 fs was used to integrate the motion equations in the simulation processes. Energy minimization by the steepest descent algorithm was first carried out after model construction to avoid poor contact, followed by isothermal–isobaric equilibrium at 298.15 K/1 bar for 50 ps. Production runs were then carried out under NPT ensemble (constant number of atoms, constant temperature with the Nose–Hoover thermostat and constant pressure with the Parrinello–Rahman barostat) for 20 ns. Three–dimensional periodic boundary conditions were used in all simulations.

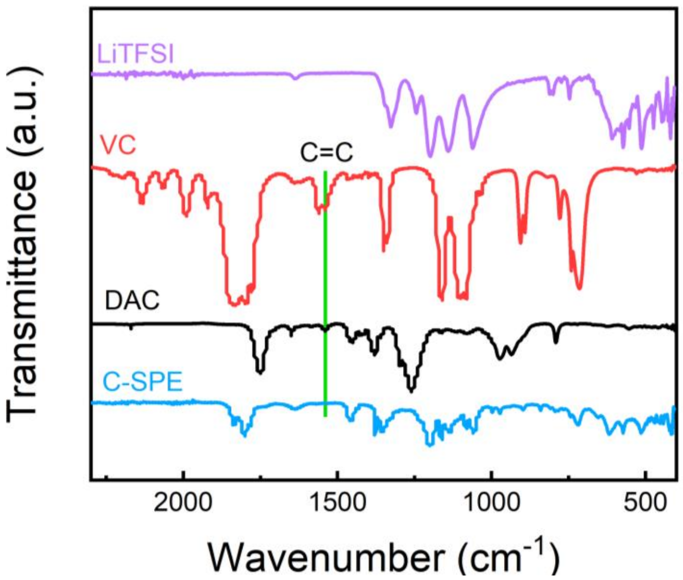

Figure S1. FTIR spectra of LiTFSI, VC, DAC and C-SPE.

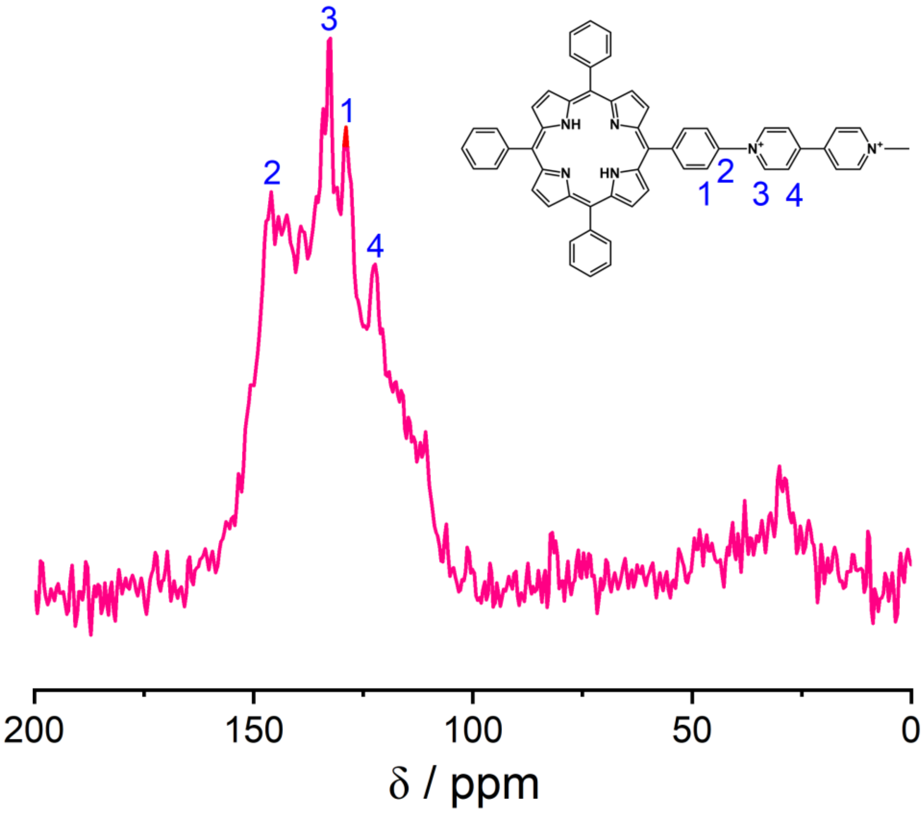

Figure S2. Solid–State ^{13}C NMR spectrum of cationic COF.

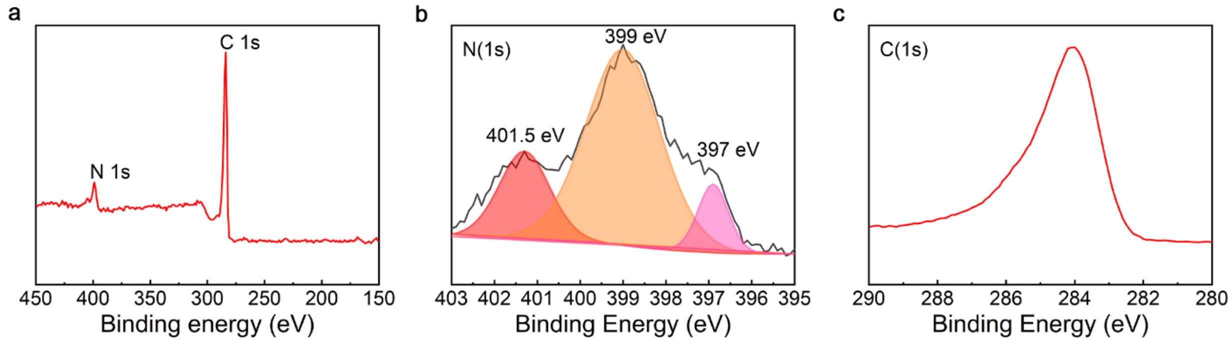

Figure S3. The XPS spectra of cationic COF. (a) XPS spectrum, (b) N 1s spectrum, (c) C 1s spectrum.

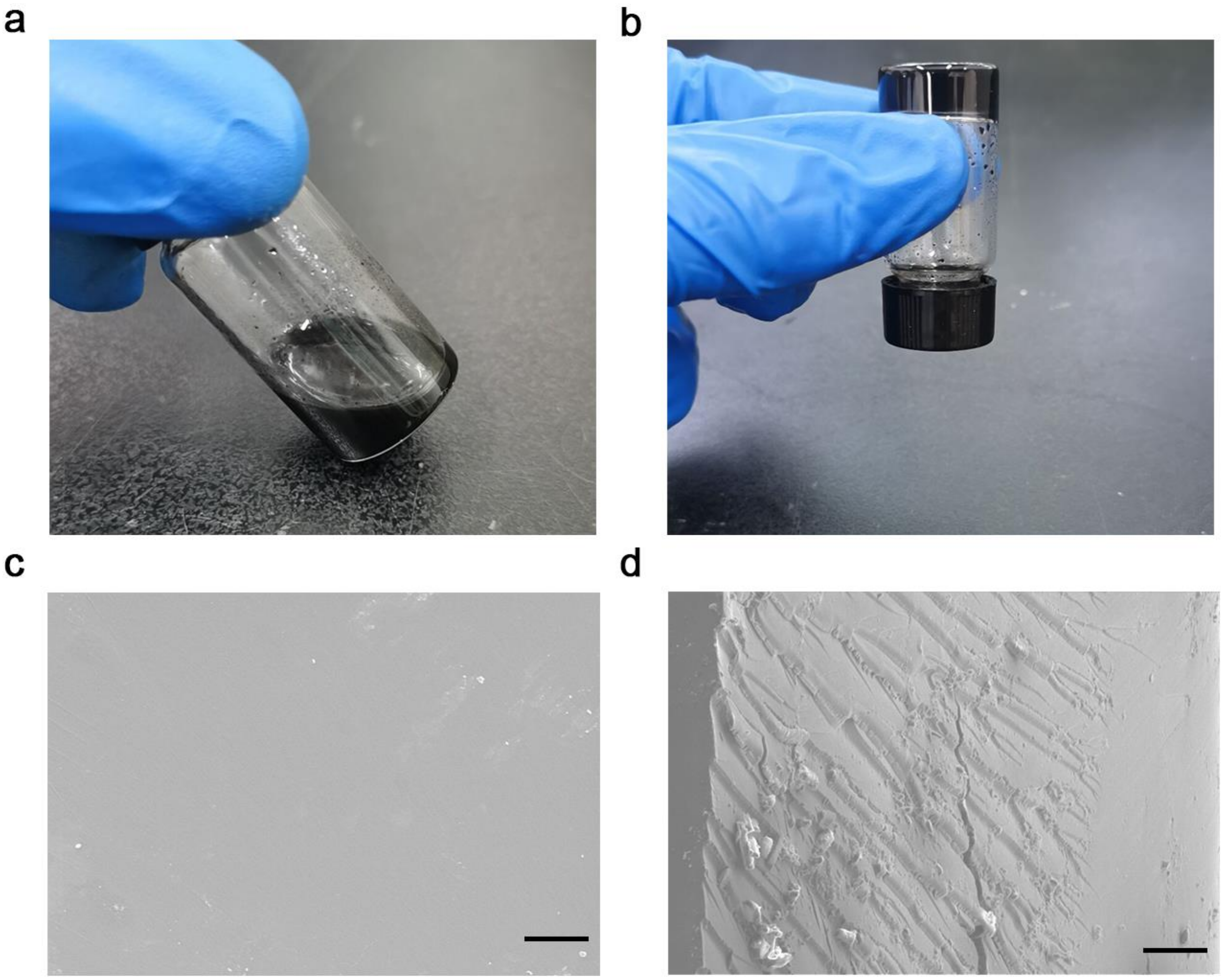

Figure S4. The image of C-SPE (a) before and (b) after polymerization. SEM of C-SPE from the (c) top–view and (d) cross–section. Scale bars: 50 µm.

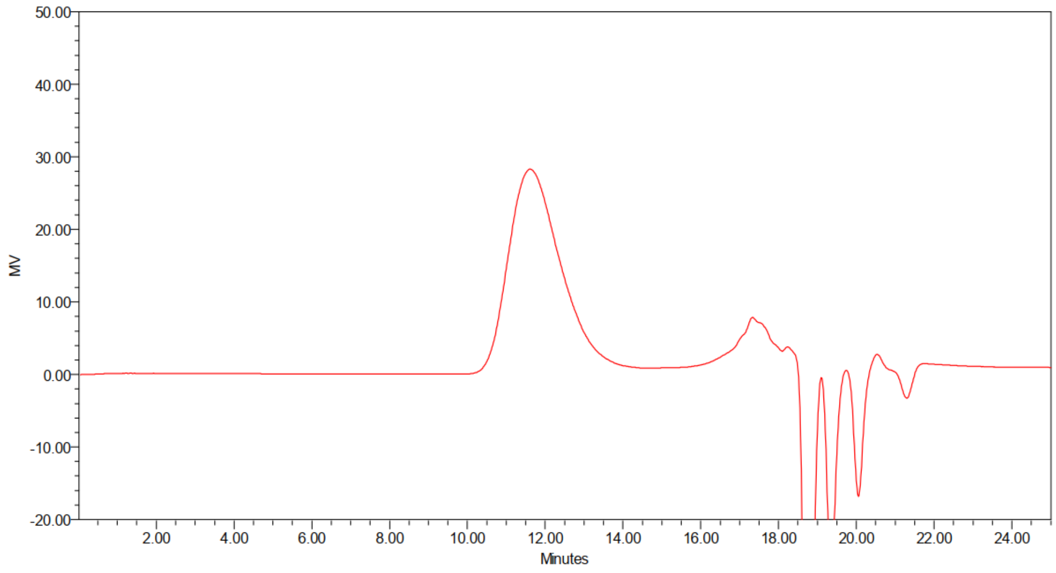

Figure S5. GPC result of PDV.

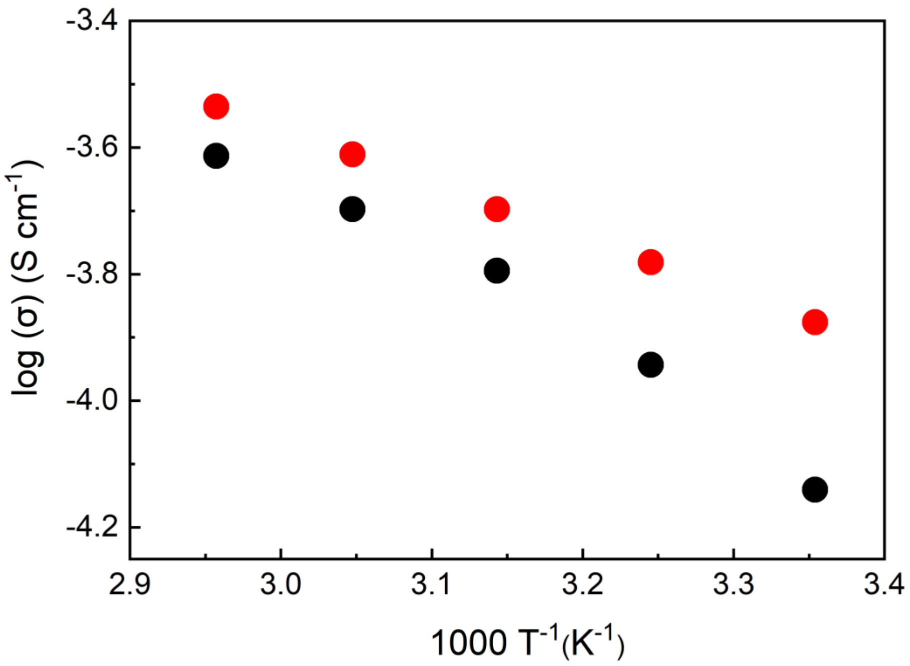

Figure S6. The ionic conductivity of C-SPE and P-SPE at temperature range from 25 °C to 65 °C (Red dot: C-SPE, Black dot: P-SPE).

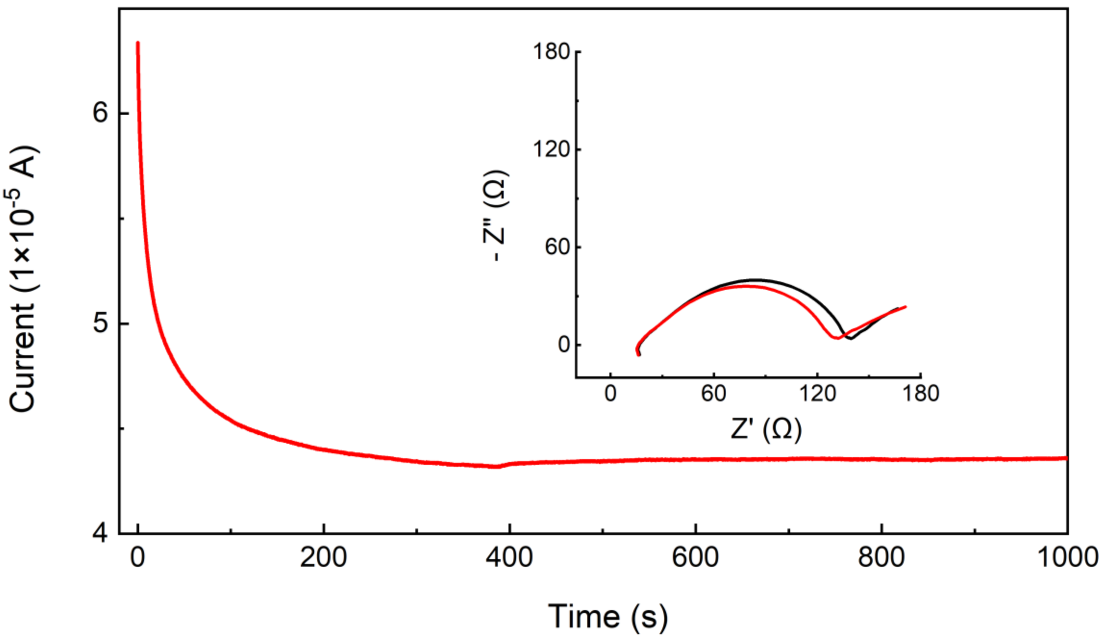

Figure S7. Li ion transform number (t_+) of P-SPE. Insert is EIS curves before (red) and after (black) polarization.

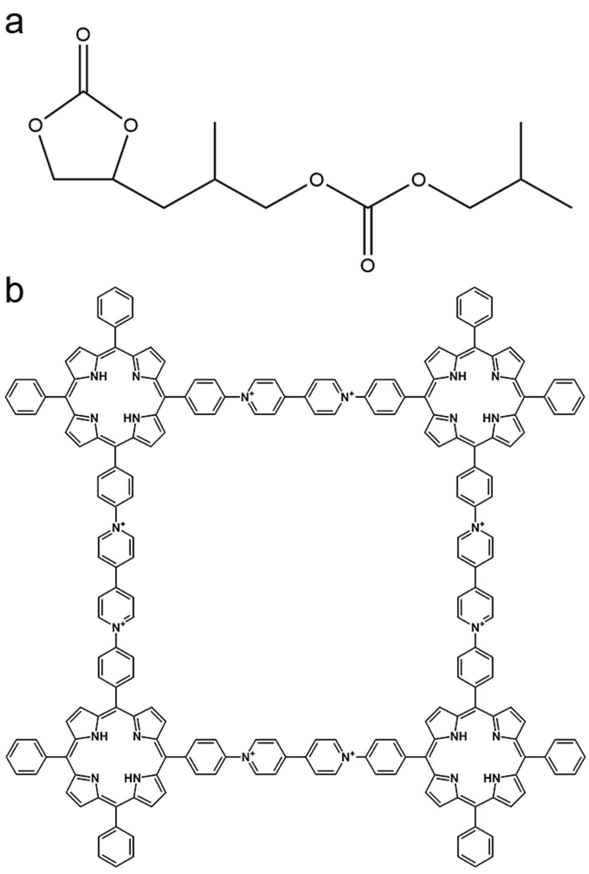

Figure S8. Structural units of (a) PDV, (b) Cationic COF used in DFT calculations.

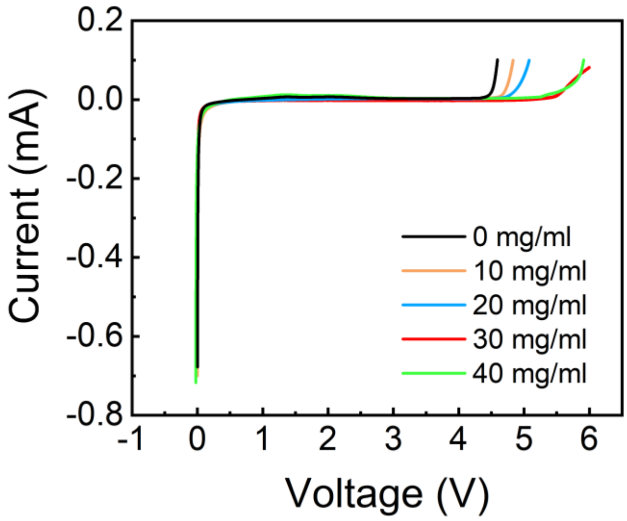

Figure S9. Electrochemical stability windows of C-SPE with different amount of COF.

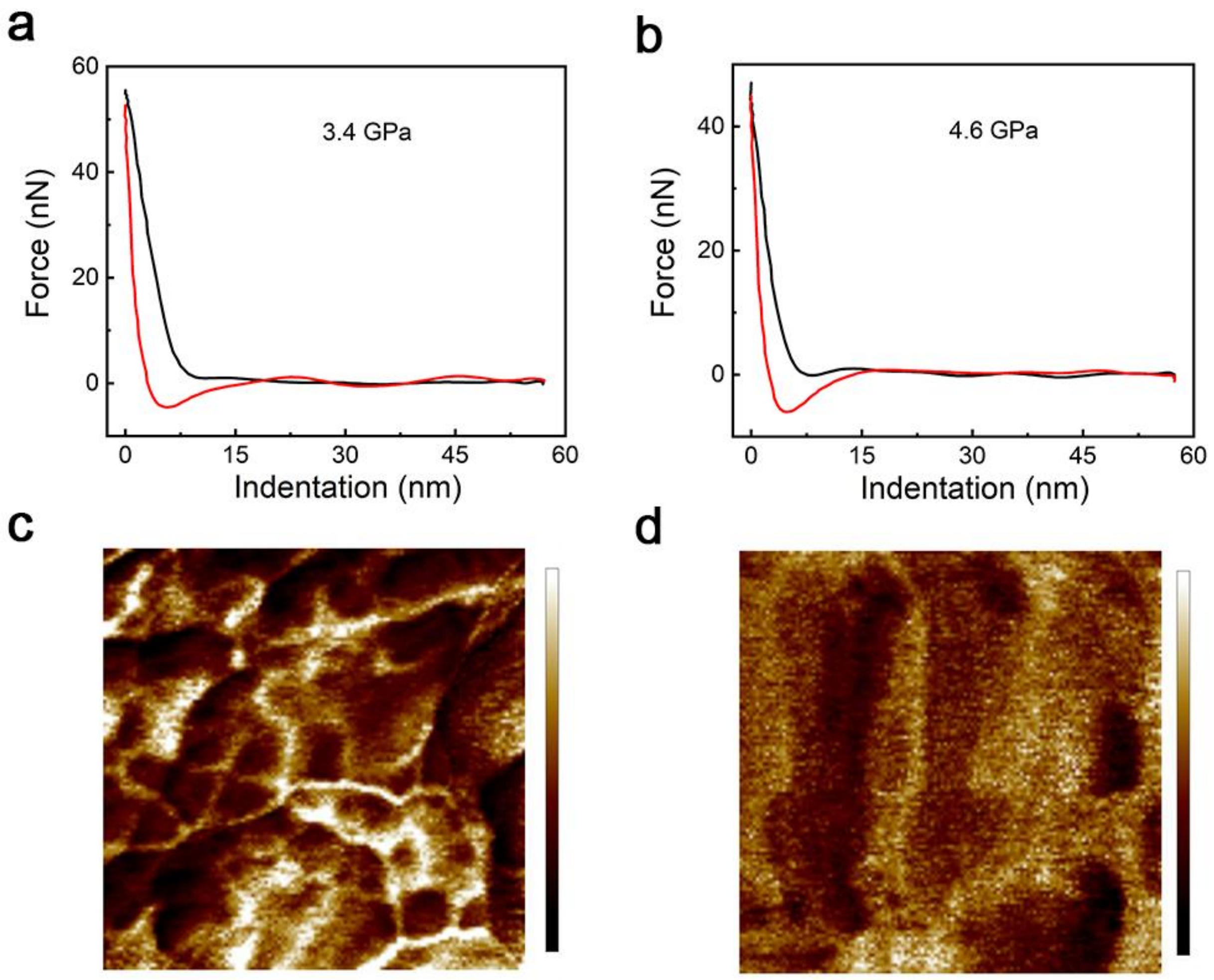

Figure S10. The typical force–displacement curve of (a) P-SPE and (b) C-SPE (Black: Extend curve, Red: Retract curve). The Young's modulus map of (c) P-SPE (scan size: 0.5 × 0.5 μm^2, scale bar: 0.6-6.8 GPa) and (d) C-SPE (scan size: 0.5 × 0.5 μm^2, scale bar: 1.9-8.6 GPa).

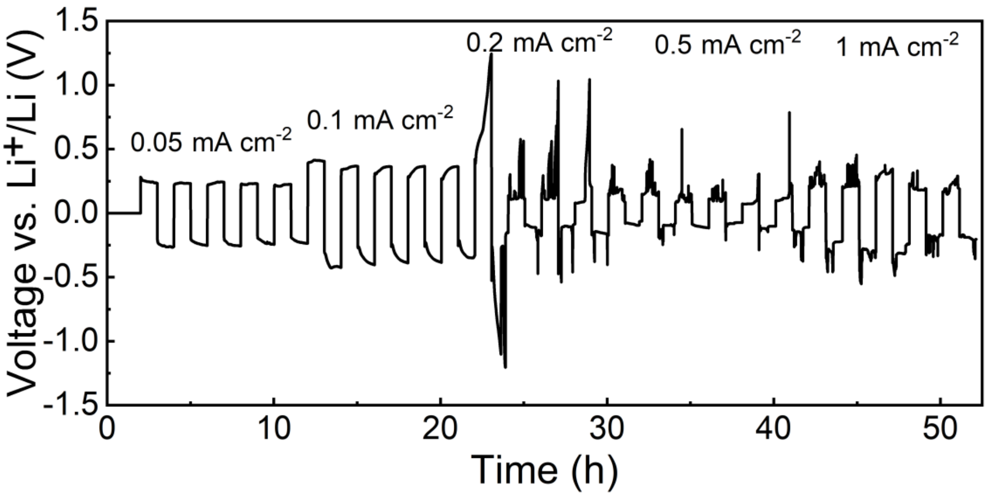

Figure S11. The voltage profiles of lithium plating and stripping in Li|Li cell with P-SPE at different current density.

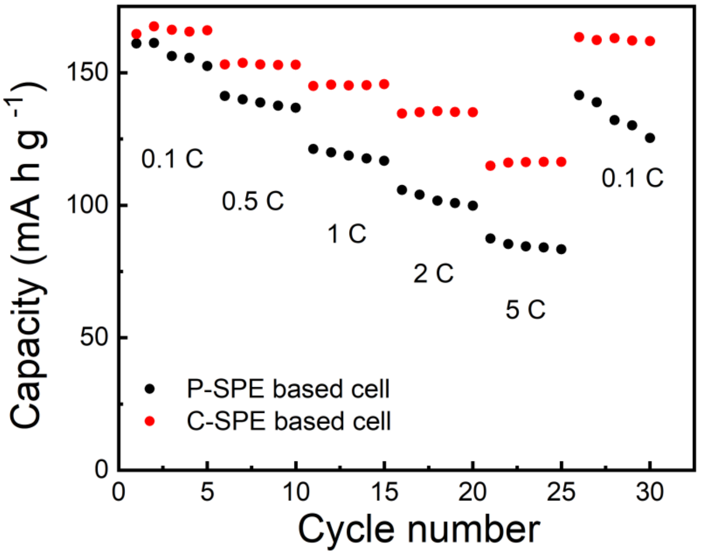

Figure S12. The rate performance of the C-SPE based cell and P-SPE based cell.

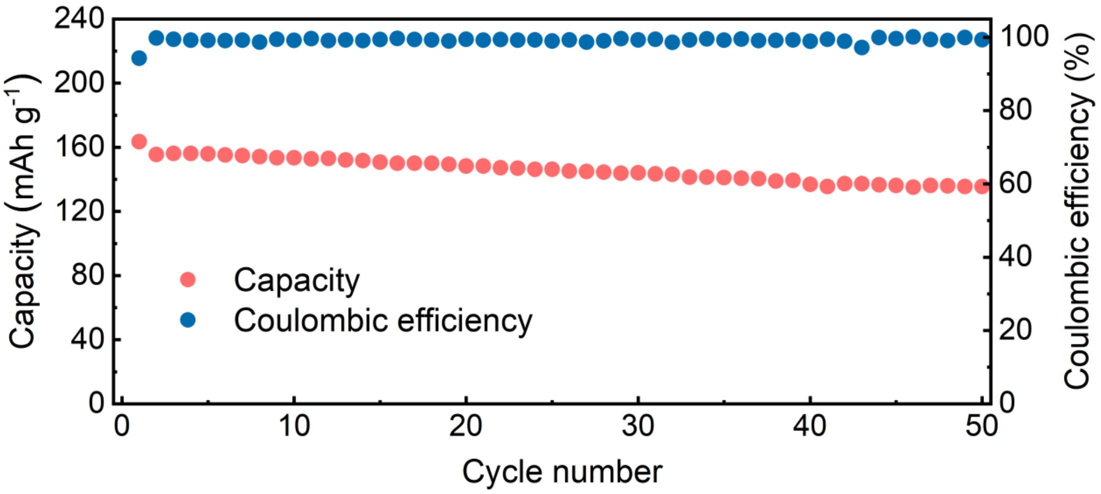

Figure S13. Cycling performance of $LiCoO_2$|C-SPE|Li cell at the current density of 0.1C.

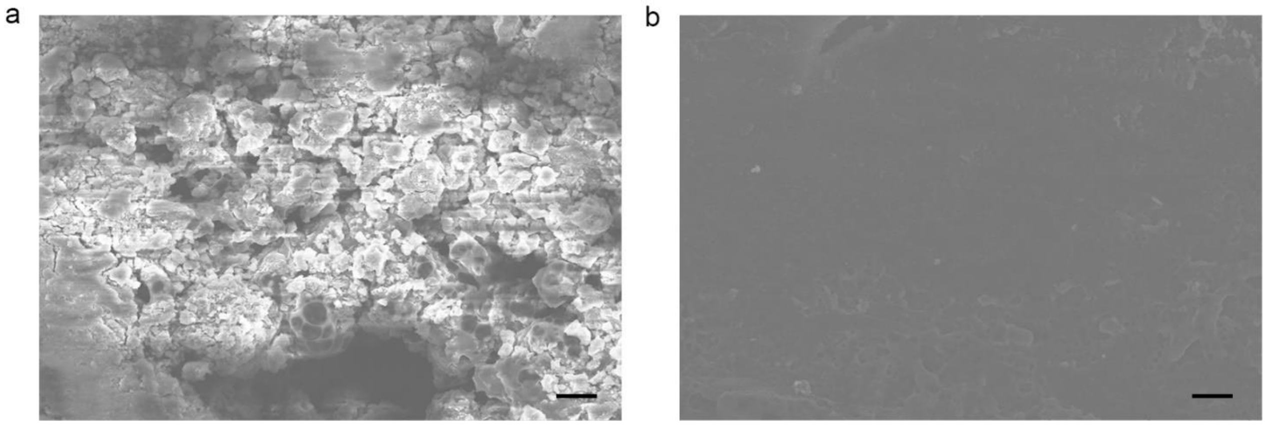

Figure S14. SEM image of Li metal surface of (a) P-SPE cell and (b) C-SPE cell at 1C after 200 cycles. Scale bars: 10 µm.

Table S1. Ionic conductivity values of P-SPE with the different weight ratios of VC/DAC.

Weight ratio of VC/DAC	Ionic conductivity (S cm^{-1})
5:1	5.8×10^{-5}
4:1	6.3×10^{-5}
3:1	7.2×10^{-5}

Table S2. A comparison table on the electrochemical performances of different solid electrolytes.

Electrolyte	Battery system	Windows (V vs Li^+/Li)	Conductivity (S cm^{-1})	Cycling performance	Ref.
Poly(Vinylene carbonate)	$LiCoO_2$/Li (2.5-4.3V)	4.5 (50℃)	9.82×10^{-5} (50℃)	84.2% after 150 cycles, at 0.5C	7
Poly(Ethylene glycol) diacrylate	$LiCoO_2$/Li (2.5-4.3V)	4.56 (RT)	8.81×10^{-3} (30℃)	87% after 100 cycles, at 0.5C	8
Poly(Ethylene carbonate)	$LiFePO_4$/Li (2.5−4 V)	4.5 (RT)	1×10^{-7} (RT)	100 mAh g^{-1}, at C/15 (75℃)	9
Poly(Propylene carbonate)	$LiFe_{0.2}Mn_{0.8}PO_4$/Li (2.5−4.35V)	4.5 (RT)	1.25×10^{-3} (RT)	90% after 70 cycles, at 0.1C	10
Poly(Succinonitrile)	$LiFePO_4$/Li (2.5−4 V)	4.6 (RT)	7.02×10^{-4} (RT)	154.4 mAh g^{-1}, at 0.1C	11
Poly(Urethane)	$LiFePO_4$/Li (2.5−4 V)	4.5 (RT)	1.9×10^{-4} (RT)	90% after 100 cycles, at 0.1C	12
Poly(Ethylene oxide)	$LiFePO_4$/Li (2.5−4 V)	4.0 (70℃)	3.79×10^{-4} (90℃)	146 mAh g^{-1}, at 0.1C	13
Poly(Tetrahydrofuran)	$LiFePO_4$/Li (2.5−4 V)	4.8 (RT)	1.2×10^{-4} (RT)	129 mAh g^{-1}, at 0.1C	14
Poly(Vinylene carbonate-diallyl carboante)	$LiCoO_2$/Li (3−4.5 V)	5.2 (RT)	1.3×10^{-4} (RT)	83.9% after 200 cycles, at 1C	This work

REFERENCE

(1) C. -L. Sun, L. -P. Liu, F. Tian, F. Ding, L. -W. Wang, *Phys. Chem. Chem. Phys.* **2018**, *20*, 23301.

(2) W. L. Jorgensen, D. S. Maxwell, J. Tirado–Rives, *J. Am. Chem. Soc.* **1996**, *118*, *45*, 11225–11236.

(3) L. S. Dodda, I. Vaca, J. Tirado–Rives, W. L. Jorgensen, *Nucleic Acids Res.* **2017**, *45*, *W1,* W331–W336.

(4) Y. Sun, J. Zhou, H. Ji, J. Liu, T. Qian, C. Yan, *ACS Appl. Mater. Interfaces.* **2019**, *11*, *35*, 32008–32014.

(5) A. K. Rappé, C. J. Casewit, K. Colwell, W. A. Goddard III, W. M. Skiff, *J. Am. Chem. Soc.* **1992**, *114, 25*, 10024-10035.

(6) T. Lu, F. W. Chen, *J. Comput. Chem.* **2012**, *33,* 580–592.

(7) J. Chai, Z. Liu, J. Ma, J. Wang, X. Liu, H. Liu, J. Zhang, G. Cui, L. Chen, *Adv. Sci.* **2017**, *4*, 1600377.

(8) M. Liu, Y. Wang, M. Li, G. Li, B. Li, S. Zhang, H. Ming, J. Qiu, J. Chen, P. Zhao, *Electrochim. Acta.* **2020**, *354*, 136622.

(9) K. Kimura, H. Matsumoto, J. Hassoun, S. Panero, B. Scrosati, Y. Tominaga, *Electrochim. Acta.* **2015**, *175,* 134.

(10) X. Y. Huang, J. Y. Huang, J. F. Wu, X. Y. Yu, Q. Z. Gao, Y. Luo, H. Hu, *RSC Adv.* **2015**, *5,* 52978.

(11) K. Liu, F. Ding, J. Liu, Q. Zhang, X. Liu, J. Zhang, Q. Xu, *ACS Appl. Mater. Interfaces.* **2016**, *8,* 23668–23675.

(12) N. Wu, Y. R. Shi, S. Y. Lang, J. M. Zhou, J. Y. Liang, W. Wang, S. J. Tan, Y. X. Yin, R. Wen, Y. G. Guo. *Angew. Chem.* **2019,** *131,* 18314–18317.

(13) Q. Ma, H. Zhang, C. Zhou, L. Zheng, P. Cheng, J. Nie, W. Feng, Y-S. Hu, H. Li, X. Huang, L. Chen, M. Armand, Z. Zhou, *ACS Appl. Mater. Interfaces.* **2016**, *8,* 29705–29712.

(14) D. G. Mackanic, W. Michaels, M. Lee, D. Feng, J. Lopez, J. Qin, Y. Cui, Z. Bao, *Adv. Energy Mater.* **2018**, *8,* 1800703.
